# The Molecular Epidemiology of Epizootic Hemorrhagic Disease Viruses Identified in Israel between 2015 and 2023

**DOI:** 10.3390/epidemiologia5010006

**Published:** 2024-02-20

**Authors:** Natalia Golender, Bernd Hoffmann

**Affiliations:** 1 Department of Virology, Kimron Veterinary Institute, Bet Dagan 5025001, Israel; 2 Institute of Diagnostic Virology, Friedrich-Loeffler-Institut, 17493 Greifswald-Insel Riems, Germany; bernd.hoffmann@fli.de

**Keywords:** cattle, arbovirus, *Reoviridae*, *Orbivirus*, ruminants, sequencing, phylogeny, outbreaks

## Abstract

Epizootic hemorrhagic disease (EHD) is an infectious, non-contagious viral disease seriously affecting cattle and some wild ruminants and has a worldwide distribution. All viruses can be subdivided into “Eastern” and “Western” topotypes according to geographic distribution via the phylogenetic analysis of internal genes. In Israel, during the last decade, three outbreaks were registered: caused by EHDV-6 in 2015, by EHDV-1 in 2016, and by EHDV-7 in 2020. Additionally, RNA of EHDV-8 was found in imported calves from Portugal in 2023. During the same period in other countries of the region, non-Israeli-like EHDV-6 and EHDV-8 were identified. Full genome sequencing, BLAST, and phylogenetic analyses of the locally and globally known EHDV genomes allowed us to presume the probable route and origin of these viruses detected in Israel. Thus, EHDV-6 has probably been circulating in the region for a long period when EHDV-1 and -8 appeared here for the last years, while their route of introduction into the new areas was probably natural; all of them belonged to the “Western” topotype. In contrast, EHDV-7 probably had the “Eastern”, anthropogenic origin. Data from the study can facilitate the evaluation of the appearance or reappearance of EHDVs in the Mediterranean area and enhance the planning of prevention measures.

## 1. Introduction

Epizootic hemorrhagic disease (EHD) is an infectious, non-contagious viral disease that is transmitted by blood-sucking insects of the genus *Culicoides*. This virus belongs to the genus *Orbivirus* of the family *Sedoreoviridae* [1,2]. The virus has a dsRNA linear genome of ten segments, which coded for seven structural (VP1-VP7) and three or four non-structural (NS1-NS3 and NS3a) proteins [3,4,5]. It shares many morphologic and structural characteristics with other members of the genus, such as the Bluetongue virus (BTV), African horse sickness virus, and equine encephalitis virus, and demonstrates immunological cross-reactivity with the Bluetongue virus group [6]. Like BTV, the primary determinant of serotype specificity is the outer capsid VP2 protein [7]. There are seven officially determined serotypes [8] and at least three putative strains representing new EHDV serotypes [9,10,11].

EHD was first described in white-tailed deer (*Odocoileus virginianus*) in New Jersey in 1955 [12], which are especially susceptible and the most affected ruminant species by EHDV, with high morbidity and mortality rates. Although other ruminant species, such as pronghorn, mule deer, and black-tailed deer, may develop clinical signs, most EHDV infections of ruminants are mild or subclinical [13].

Previously, EHDV was thought to cause asymptomatic infection in cattle [14], except for the EHDV-2 Ibaraki strain, which was responsible for an extensive outbreak of the disease in cattle in Japan during 1959 [15,16,17,18].

However, EHDV-6 and 7 were responsible for the clinical manifestation of the disease in the Far and Middle East and North Africa. EHDV-6, which was observed in 2006 in Tunisia, in 2007 in Turkey, and in 2015 in Japan and Israel. The infected cattle showed substantial clinical signs, which included fever, milk reduction, edema of the head, necrotic lesions of the oral mucosa, hypersalivation, hyperthermia, lesions of the teats, stiff gate, dysphagia and cessation of rumination, and redness of the lips and muzzle, accompanied by anorexia and respiratory distress [10,19,20,21,22,23]. Notably, epizootic diseases caused by EHDV-7 in 1997 in Japan were mainly characterized by abortion and stillbirth in addition to febrile illness [24], while for EHDV-7 in Israel, the clinical signs resembled those which were caused by EHDV-6 [22,25]. Successful virus isolation (VI) was also carried out from aborted fetuses and placentas [18], suggesting that the blood–placental barrier was crossed and the fetus was infected. Similarly, viral RNA was also detected in the aborted fetuses and placentas of Israeli cattle affected by EHDV-6 [22].

Comparing EHD caused by serotype 1, this serotype was not associated with clinical manifestation in ruminants in Japan [26], whereas Israeli EHDV-1 caused mostly asymptomatic or mild infection in cattle [27]. Considering other EHDV clinically described serotypes/strains, EHDV-10 was isolated from asymptomatic cattle in Japan [19], while novel Chinese EHDV was observed in a single febrile sentinel calf [11]. In contrast to the above-mentioned EHDV serotypes, recently identified EHDV-8 in the Mediterranean region is characterized by erosions on teats and mucosal membranes, cyanosis and edema of the tongue, submandibular edema, conjunctivitis, conjunctivitis and lacrimation, nasal discharge, respiratory distress, inappetence, and fever [28,29].

EHDV has a worldwide distribution. In East Asia, at least seven serotypes of EHDV (EHDV-1, 2, 5, 6, 7, 8, and 10) have been discovered [10,19,30,31,32]. In Western Asia (the territory of Bahrain, Oman, and Israel), four serotypes of EHDV were identified (EHDV-1, -2, -6, and -7) [22,25,27,33,34]. In Turkey, which crosses Europe and Asia, an outbreak in cattle in goitered gazelle caused by EHDV-6 during 2007 was reported [21,35]. At least five eastern serotypes of EHDV (EHDV-2, -5, -6, -7, and -8) were isolated in Australia [4,33,36]. EHDV-1, -2, and -6 have wildly spread in the United States and Canada, where the disease was reported in deer and cattle [37,38,39,40,41]. In South America, EHDV-1 was isolated, and EHDV-6 was detected in both French Guiana and Ecuador [42,43]. Regarding the African continent, EHDV-1 and EHDV-6 were identified, but EHDV-6 and EHDV-8, which recently caused outbreaks in cattle, are more common [28,44,45,46,47]. The only strain of EHDV-4 in the world was discovered in Nigeria [33]. EHDV-8 was the first serotype reported on the territory of Europe (in Sardinia and Sicily, Italy, Spain, Portugal, and France) [29].

The novel history of EHDV in the “old world” began in 2003, when EHDV-6 was registered in Reunion Island [48]. A few years later, large outbreaks caused by EHDV-6 were reported in several countries of the Mediterranean Basin, including Morocco, Algeria, and Tunisia in 2006, and in the following year in Turkey [21,24,48]. Antibodies against EHDV-6 were identified in sera samples collected in 2012–2013 in Tunisia [45], illustrating the continuous circulation of the virus in the region, which was confirmed by the evidence of a low seroprevalence among samples collected in 2015 in Libya [46]. Regarding other parts of Africa, RT-qPCR EHDV-positive samples were detected in the field samples collected from cattle between 2007 and 2010 in Kenya [49]. Recently, EHDV-6 was detected in asymptomatic cattle in 2016 in Mayotte [50] and caused an outbreak in 2015 in Japan [10,19] and Tunisia [51]. The latest EHD outbreaks caused by serotype 8 were reported during 2021–2023 in the Mediterranean region, including in Tunisia, Italy, Spain, Portugal, and France [28,52].

In Israel, EHD was suspected in 1951 [53] but was first confirmed as an EHDV-7 outbreak in 2006 [24]. The next outbreak, caused by EHDV-6, was observed in 2015 [23]. During the following year (2016), EHDV-1 was identified in Israeli cattle and a wild mountain gazelle [27]. Notably, in the same year, EHDV-1 also was detected in Egypt [54]. The last outbreak of EHD in Israel was observed in 2020 when the Eastern genotype of serotype 7 was identified. The latest identification of the EHDV viral RNA was carried out in collected whole blood samples from imported Portuguese calves in September 2023. Due to the specific geographic situation of Israel, the local ruminant population has frequently been exposed to different serotypes and strains of EHDV, which are genetically and clinically different. The aim of the current work is a description of the probable routes of EHDV-7 introduction into Israel and its spread during 2020 outbreak based on phylogenetic analysis and collected epidemiological data.

## 2. Materials and Methods

### 2.1. Field Samples

A total of 373 samples from 367 animals collected in 2020 from ill and dead cattle and wild/zoo ruminants, including aborted or malformed domestic or wild ruminant fetuses, were submitted for routine examination to the virology department of the Kimron Veterinary Institute, Israel (KVI). In addition, 635 whole blood samples from cattle tested in 2021 and 1178 tested samples collected in 2023 were included in this study. Since no EHDV-positive samples were identified in 2022, data on these samples were not included in this work. Clinical specimens collected in 2020 included the placenta, brain, and internal organs from aborted fetuses, whole blood from symptomatic ruminants, and spleen or lung from dead ruminants. Data on field samples tested for EHDV in 2020–2021 and 2023 are summarized in Table 1.

### 2.2. Nucleic Acid Extraction and Pan-EHDV Real-Time Polymerase Chain Reaction (RT-PCR)

We extracted ribonucleic acid (RNA) from the tissue culture supernatant, chicken embryo homogenates, and field samples (whole blood, lung, and spleen) using the Invisorb Spin Virus RNA Mini Kit (STRATEC Molecular GmbH, Berlin, Germany), MagMAX™ CORE Nucleic Acid Purification Kit (Thermo Fisher Scientific, Austin, TX, USA), and IndiMag Pathogen Kit (Indical Bioscience, Leipzig, Germany). Viral RNA detection was performed using the VetMAX™ EHDV kit (Applied Biosystems™, Thermo Fisher Scientific Inc., Lissieu, France). The pan-EHDV system described by Wernike et al. [55], which is based on the detection of Seg-5 fragment, was used as an alternative method. In accordance with the instructions of the authors and manufacturer of the RT-qPCR kit/system, the cut-off for all these methods was Cycle Threshold (Ct) 40.

### 2.3. Type-Specific RT-PCR and Sanger Sequencing

For the identification of EHDV-8 by sequencing, the conventional RT-PCR was performed using a One-Step RT-PCR kit (Qiagen, Hilden, Germany); data on primers based on the Seg-2 detection of EHDV-6 and 8 are provided in Table 2. Primers were developed using the Genius 9.05 program (Biomatters Ltd., Auckland, New Zeeland) when EHD6/8-1F and EHD6/8-250R were designed to recognize EHDV-6 and EHDV-8; EHD8-S2-178F and EHD8-S2-522R were developed specifically to recognize a recently emerged EHDV-8; and EHD8-S2-447F and EHD8-S2-705R primers were developed to detect the Australian strain of EHDV-8 (Table 2). Primers for the partial sequencing of internal genes of EHDV-7 and primers used for covering missed regions from the sequence from whole genome sequencing (WGS) of EHDV-7 are shown in Table S1. The cDNA fragments of positive samples were purified using the MEGAquick-spin Total Fragment DNA Purification Kit (iNtRON Biotechnology, Gyeonggi-do, South Korea) and subsequently sequenced by standard Sanger methods in both directions using an ABI 3730xl DNA Analyzer (Hylabs, Rehovot, Israel).

### 2.4. Whole Genome Sequencing and Phylogenetic Analysis

Extracted RNA of the EHDV-7 strain ISR-2262/2/20 was submitted to Genotypic Technology Pvt. Ltd., Bangalore, India. The whole procedure was described previously [56,57]. The resulting nucleotide (nt) sequences of the EHDV-7 and -8 were assembled, and nt sequences were aligned and pairwise compared using Geneious version 9.0.5 (Biomatters, Auckland, New Zealand) and/or BioEdit programs (https://bioedit.software.informer.com/7.2/ (accessed on 9 March 2017). 

Using HTS-SISPA technology [58] and the previously described procedure [59] (Ries 2020), the double-sense (ds) cDNA of EHDV-6 strain ISR-4487/15 and EHDV-1 strain ISR-2096/16 were prepared and submitted to Eurofins Genomic (Ebersberg, Germany) for genome sequencing on the Illumina platform. The obtained fastq raw data were further processed using the Geneious Prime v2021.0.1 software (Biomatters Ltd., Auckland, New Zeeland) to construct complete genome sequences of the EHDV-6 strain ISR-4487/15 and EHDV-1 strain ISR-2096/16.

Phylogenetic trees were constructed using the Mega X software [59]. For all phylogenetic trees, the maximum-likelihood method (ML) and the Tamura–Nei models were applied. The sequences of the Israeli ISR-2096/15 (EHDV-6), ISR-4487/16 (EHDV-1), ISR-2262/2/20 (EHDV-7), ISR-1692/2/23, and ISR-1692/9/23 (EHDV-8) strains were used as a representative strain for all phylogenetic trees of the phylogenetic analyses. As an outgroup BTV, different strains were chosen based on the BLAST NCBI analysis of one of the most closely related viral species to EHDV by internal genes, except Seg-6, where closely related BTV were not suitable as an outgroup due to clustering with EHDV-4 and -5 strains. For this purpose, the Warrego virus (WARV) was used.

### 2.5. Virus Isolation

Attempts to isolate EHDV directly on Vero (African green monkey kidney) and BHK-21 (baby hamster kidney) cells failed. Thus, red blood cells (RBS) were washed three times in PBS. The washed RBS were disrupted via dilution in sterile double-distilled water in a proportion of 1:10 (RBS to water). The resulting solution was used for inoculating Vero and BHK-21 cells, incubated in the cell incubator for one hour at a temperature of 37 °C, and washed two times with PBS. Eagle’s Minimum Essential Medium (EMEM), supplemented with 2% fetal bovine serum (FBS) and 1% penicillin–streptomycin (10,000 U/mL), was added to the cell monolayer. Cells were observed daily for the appearance of cytopathic effects. Three blind passages were performed. Sixty-two samples positive in RT-qPCR for EHDV samples were inoculated into embryonated chicken eggs (ECE) according to the method described by Komarov and Goldsmit [55].

## 3. Results

### 3.1. Clinical Signs in Affected Animals and Geographic Distribution of Israeli EHDVs

Clinical signs in affected animals and the geographic distribution of EHDV-6 and EHDV-1 were previously described. In brief, the main clinical signs observed in EHDV-6-affected cattle included reduced milk production, weakness, drooling, lameness and recumbency, fever, slight erythema of nasal and oral mucosae, weight loss, and abortion. Dyspnea, cachexia, and death were observed less frequently [22]. Regarding EHDV-1-affected cattle, the clinical signs were milder, compared with those caused by EHDV-6. In many farms, EHDV-1 infection was asymptomatic or subclinical; milk yield reduction, fever, and recumbency were the only prominent clinical signs that were seen during the outbreak [27].

Considering EHDV-7, it was first detected at Hama’apil, located in the Central District of Israel, on 23 August 2020. The next registered case was identified three weeks later at Ma’ale Gamla, the settlement located in the Western part of the Golan Heights. From mid-September to mid-October 2020, EHDV-7 was found in only six settlements—five of them situated in the North of Israel and one in the center. In the second part of October 2020, EHDV was first identified in the Southern District of Israel. Since then, it has been found in areas from the North Golan Height to the Negev desert. The last registered EHDV detections occurred in the second part of April 2021, from the settlement located in the southern part of the Central District and Negev desert, where the weather is usually warmer than in the northern part of Israel. To summarize, the virus was detected in 80 settlements. Regarding clinical signs manifested by cattle affected by EHDV-7, they included recumbency, weakness, fever, lameness, hypersalivation and nasal discharge, sporadic death in a herd, milk reduction in milking cows, diarrhea, limb edema, and abortions. In mixed with BTV cases, clinical signs included a sharp decrease in body weight up to cachexia and anemia; cases of death in a herd were also reported.

On 15 September 2023, fifteen calves without prominent clinical signs were randomly chosen for testing for EHDV and BTV by RT-qPCR from the group of calves from Portugal imported on 31 August 2023, where EHDV-8 was reported since mid-July 2023.

### 3.2. EHDV Detection by Pan-EHDV RT-qPCR from Field Samples Collected in 2020–2021 and 2023

In the 2020–2021 calendar year, 283 out of 1002 tested samples from 995 animals were positive for Pan-EHDV RT-qPCR (Table 1). In 2023, two calves, randomly selected for analysis from 15 imported calves from Portugal, were EHDV-positive and were identified by partial Sanger sequencing of Seg-2 as EHDV-8-positive. It is noteworthy that one of the imported calves was also positive in the pan-BTV-RT qPCR. Partial sequencing confirmed a high genetic homology with Spanish and Moroccan BTV-4 (data are not provided; primers for Sanger sequencing are provided in Table S1). All other samples tested in 2023 were EHDV-negative.

### 3.3. Virus Isolation

Nine EHDV-7 viruses were isolated in ECE during 2020–2021: eight from whole blood samples of viremic animals and one from a spleen sample. Five isolates from ECE were subsequently adapted to Vero or BHK-21 cells. Attempts to isolate EHDV-8 in tissue cultures and ECE were unsuccessful.

### 3.4. Sequence Analyses of EHDV-1, -6, -7, and -8

The coding regions of EHDV-1 strain ISR-2096/16, EHDV-6 strain ISR-4487/15, EHDV-7 strain ISR-2262/2/20 were completely sequenced. Complete information about the strain and data on accession numbers, which were uploaded to the INSDC, are shown in Table S2. BLAST analysis based on a comparison of nt sequences of all completely sequenced Israeli EHDV strains with global EHDV is presented in Table 3. For revealing probable ancestors, more recent viruses were not included in the study. For this reason, Israeli EHDV-6 and EHDV-1 do not show any representation of Seg-1 and -4; however, Israeli EHDV-7 from 2006, in the case of Seg-1, and EHDV-4 from Nigeria from 1968, in the case of Seg-4, were shown to have the closest relationship via BLAST analysis. Both Seg-1 of Israeli EHDV-6 and EHDV-1 have more than a 99% identity with the Israeli EHDV-7 ISR2006/04/2006 strain from 2006. To sum up, six out of ten genome segments of Israeli EHDV-6 ISR-4487/15 had a high identity with Israeli EHDV-7 strains from 2006; four of them had more than a 99% identity, probably pointing out their local, regional origin. The last four segments have a high identity with “Western” strains from Africa and the Arabian Peninsula and surrounding islands, which can indicate the co-circulation of EHDVs of African and Middle East origin in the same area. Comparing EHDV-1 to the global EHDV strains, it was seen that only Seg-1 probably has a local origin when all other segments had an African origin. Looking at the BLAST analysis of Israeli EHDV-7 ISR-2262/2/20, only Japanese and Chinese EHDV strains have high homology with Israeli EHDV-7, illustrating its “Eastern” origin. The EHDV-8 strain identified in blood samples from calves imported from Portugal showed 99.70% identity with the last published Tunisian EHDV-8 strains identified in *Culicoides* sp. in 2022 (Table 3).

### 3.5. Phylogenetic Analysis of Israeli EHDV Strains

The EHDV genome consists of 10 linear segments of double-stranded RNA. The viral genome encodes seven structural proteins (VP1 to VP7) and four different non-structural proteins (NS1, NS2, NS3/3a, and NS4).

**Figure 1:**
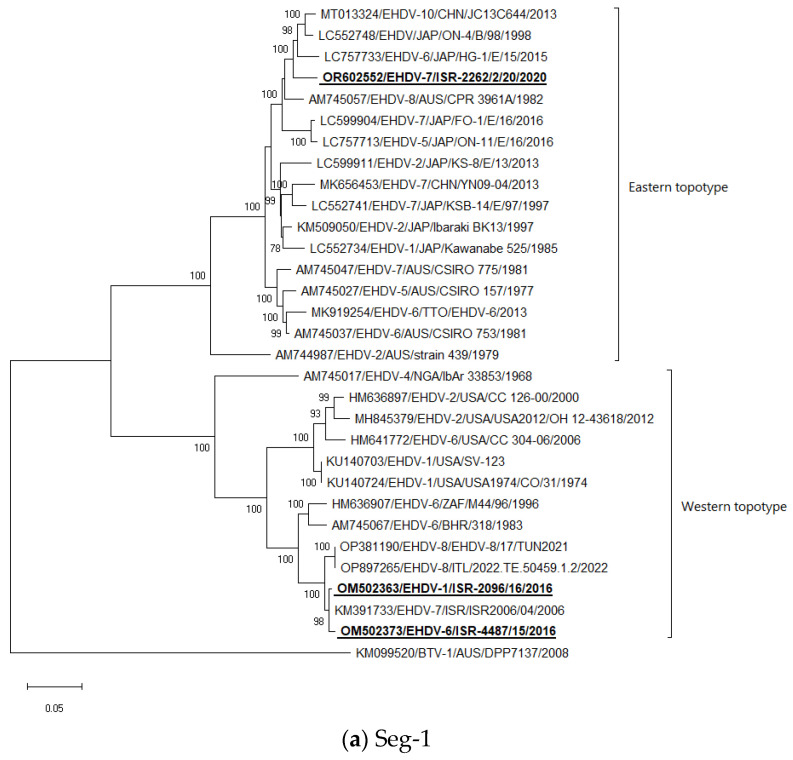

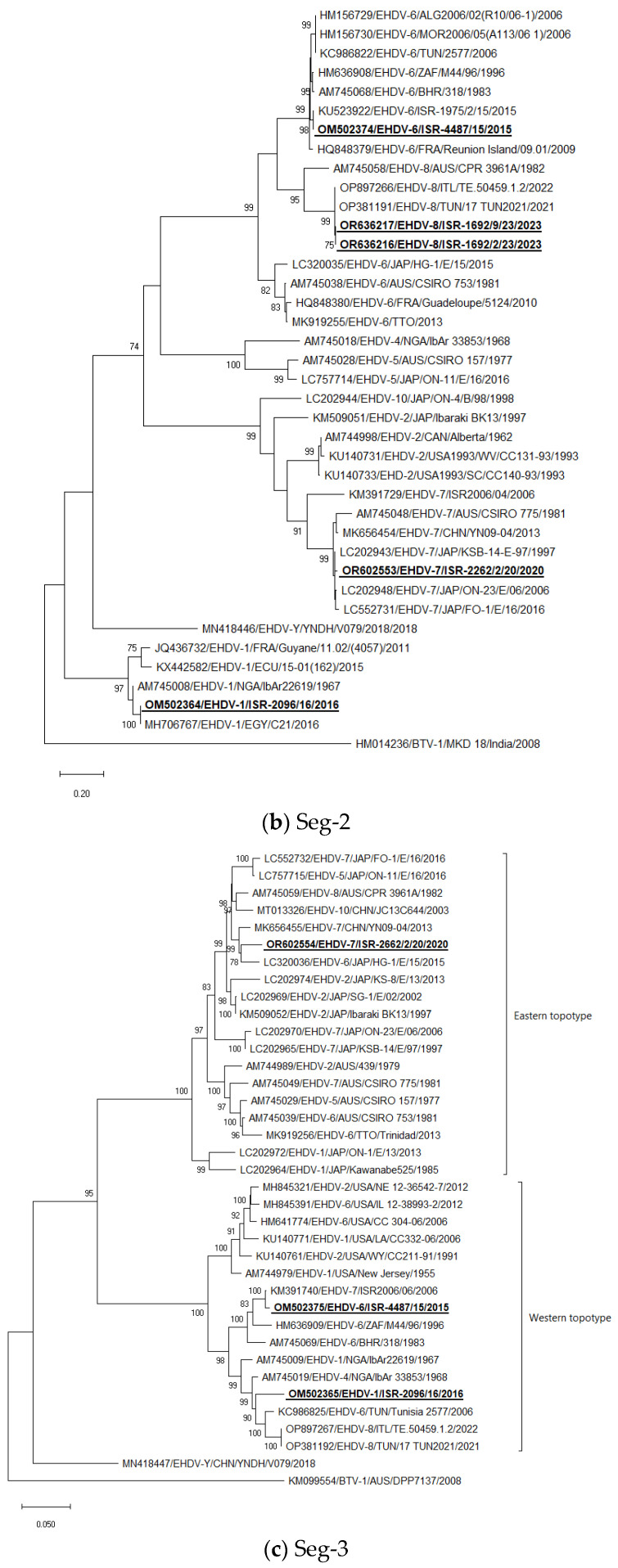

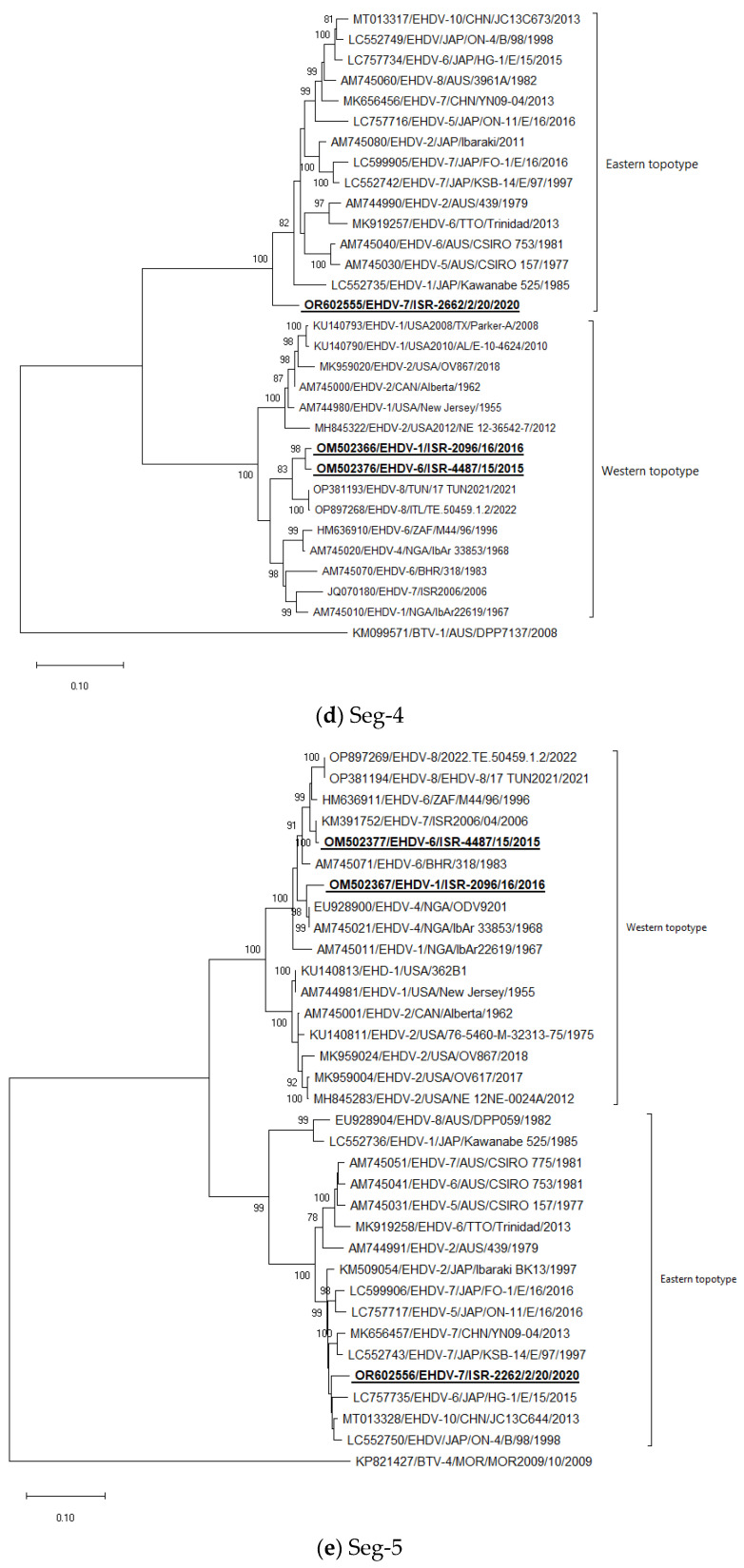

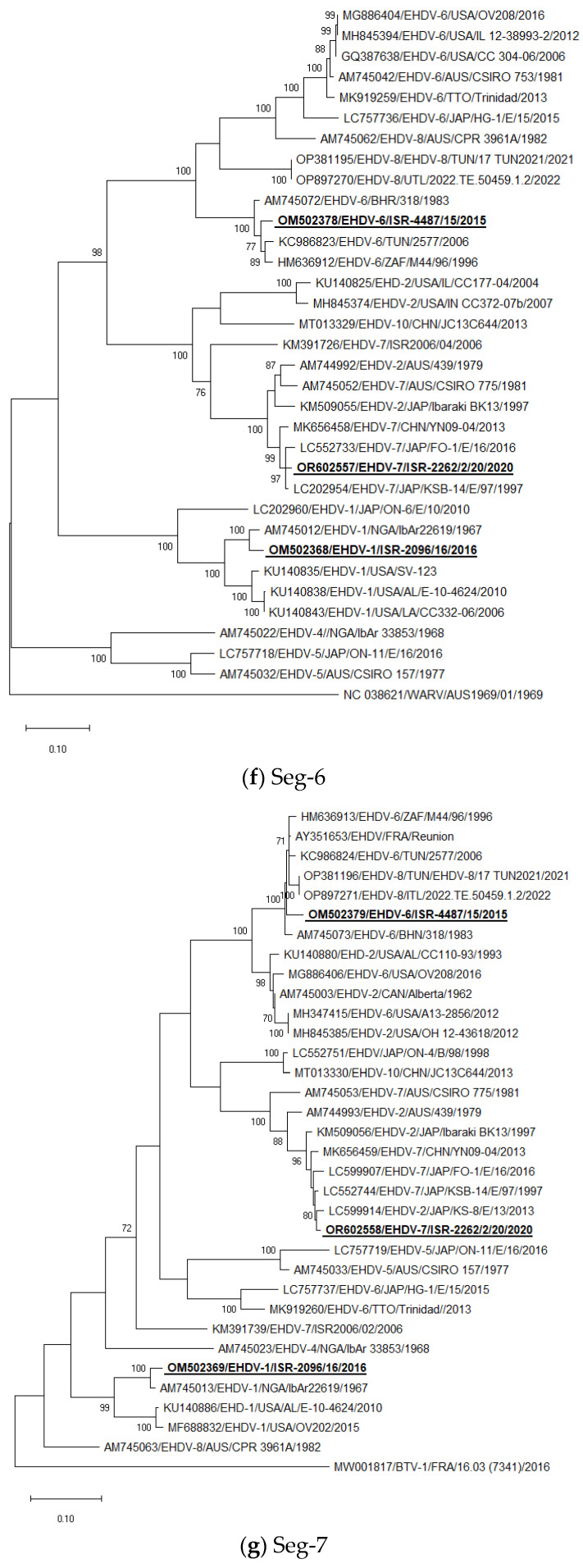

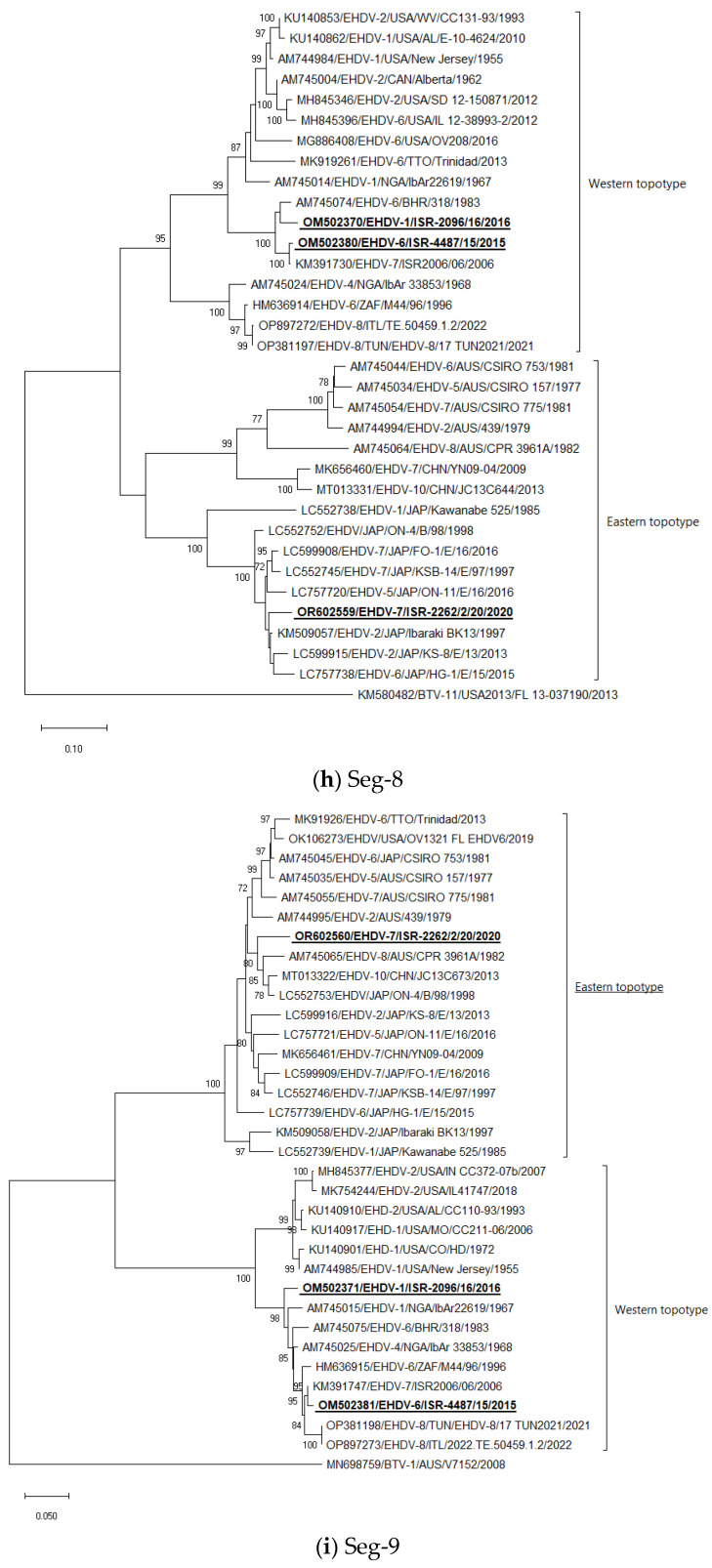

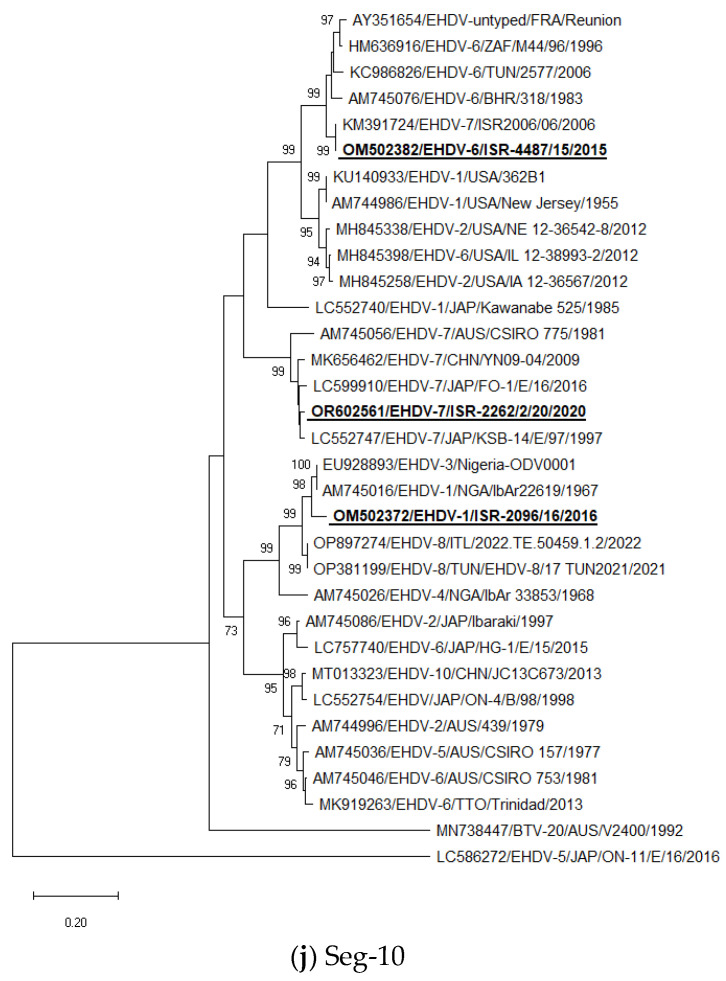
Phylogenetic trees of Israeli and global EHDV strains. (**a**–**j**) Phylogenetic trees of segments 1–10. Israeli EHDVs used in the study are shown in bold and underlined. The phylogeny was inferred using the maximum-likelihood method and the Tamura–Nei model method. The percentage of replicate trees in which the associated taxa clustered together in the bootstrap test (1000 replicates) are shown next to the branches. Viruses were identified by accession number/serotype/location/isolate/year.

Seg-1: Both Israeli EHDV-6 and EHDV-1 are most closely related to each other and to EHDV-7 from 2006. EHDV-7 from 2020 clustered with Japanese EHDV-6 strain HG-1E/15 from 2015, untyped Japanese strain ON-4/B/98 from 1998, and Chinese strain EHDV-10 strain JC13C644 from 2013 (Figure 1a).

Seg-2: Israeli EHDV-6 strains clustered with EHDV-6 strains which were identified during 2006 outbreaks in North African countries and with EHDV-6 from Bahrain, Reunion Iceland, and South Africa. Israeli EHDV-1 ISR-2096/16 clustered with EHDV-1 E21/C identified in Egypt in the same year (2016) and with Nigerian EHDV-1 IbAr22619 from 1967. The Israeli EHDV-7 strain ISR-2262/2/20 from 2020 clustered with several Japanese EHDV-7 strains. EHDV-8, which was identified in imported from Portugal calves, clustered with Tunisian, and Italian strains isolated in 2021–2022 (Figure 1b).

Seg-3: The Israeli EHDV-6 ISR-4487/15 strain clustered with the Israeli EHDV-7 ISR2006/06 strain. The EHDV-1 strain ISR-2096/16 clustered with Tunisian EHDV-6 Tunisia 2577 from 2006, and Tunisian and Italian EHDV-8 strains from 2021 to 2022. Israeli EHDV-7 ISR-2262/2/20 strain clustered with Japanese EHDV-6 strain HG-1E/15 from 2015 (Figure 1c).

Seg-4: Israeli EHDV-6 ISR-4487/15 strain clustered with Israeli EHDV-1 ISR-2096/16, forming a monophyletic group. Israeli EHDV-7 ISR-2262/2/20 strain grouped with “Eastern” strains but made a separate branch (Figure 1d).

Seg-5: The Israeli EHDV-6 ISR-4487/15 strain clustered with the Israeli EHDV-7 ISR2006/04 strain; EHDV-1 ISR-2096/16 strain clustered with Nigerian EHDV-4 strains isolated in 1968. The Israeli EHDV-7 ISR-2262/2/20 strain grouped with the following Eastern strains: Japanese EHDV-6 HG-1E/15 from 2015, untyped Japanese strain ON-4/B/98 from 1998, and Chinese strain EHDV-10 strain JC13C644 from 2013 (Figure 1e).

Seg-6: Israeli EHDV-6 strains clustered with EHDV-6 strains identified during 2006 outbreaks in North African countries and with EHDV-6 from Bahrain, Reunion Iceland, and South Africa. Israeli EHDV-1 ISR-2096/16 clustered with EHDV-1 E21/C identified in Egypt in the same year (2016) and with Nigerian EHDV-1 IbAr22619 from 1967. Israeli EHDV-7 strain ISR-2262/2/20 from 2020 clustered with several Japanese and Chinese EHDV-7 strains (Figure 1f).

Seg-7: Israeli EHDV-6 strains clustered with EHDV-6 and EHDV-8 “Western” regional strains, which originated from Bahrain, Tunisia, Italy, Reunion Iceland, and South Africa. Israeli EHDV-1 ISR-2096/16 clustered with Nigerian EHDV-1 IbAr22619 from 1967. Israeli EHDV-7 strain ISR-2262/2/20 from 2020 clustered with Japanese EHDV-2 strain KS-8/E/13, which is a slightly different result from BLAST analysis when the most closely related strain was EHDV-7 KSB-14/E/97 isolated in 1997. Notably, EHDVs cannot be strictly subdivided into “Eastern” and “Western” topotypes according to phylogenetic analysis of Seg-7 sequences (Figure 1g).

Seg-8: The Israeli EHDV-6 ISR-4487/15 strain clustered with the Israeli EHDV-7 ISR2006/04 strain; the EHDV-1 ISR-2096/16 strain clustered with EHDV-6 318 strain from Bahrain. Israeli EHDV-7 ISR-2262/2/20 clustered with the Japanese EHDV-2 Ibaraki strain BK13 from 1997 (Figure 1h).

Seg-9: The Israeli EHDV-6 ISR-4487/15 strain clustered with the Israeli EHDV-7 ISR2006/06 strain; EHDV-1 ISR-2096/16 formed a separate branch with “Western” regional strains, belonged to serotypes 1, 4, 6, 7, and 8. Israeli EHDV-7 ISR-2262/2/20 clustered with “Eastern” strains belonged untyped Japanese strain ON-4/B/98 from 1998, Chinese strain EHDV-10 strain JC13C644 from 2013, and Australian EHDV-8 strain CPR 3961A isolated in 1982, but formed a separate branch (Figure 1i).

Seg-10: The Israeli EHDV-6 ISR-4487/15 strain clustered with the Israeli EHDV-7 ISR2006/06 strain; EHDV-1 ISR-2096/16 clustered with Nigerian strains belonged to serotypes 1 and 3 (strains IbAr22619 and Nigeria-ODV0001, respectively), similarly to the results of BLAST analysis. Israeli EHDV-7 ISR-2262/2/20 clustered with Japanese EHDV-7 KSB-14/E/97 from 1997. As in the case of Seg-7, EHDVs cannot be divided into “Eastern” and “Western” topotypes by Seg-10. Additionally, some Japanese strains isolated in 2015–2017 that belong to serotypes 4 and 5 have significantly different sequences of the Seg-10 from all other global EHDV strains and are represented in Figure 1j by the EHDV-5 ON-11/E/16/2016 strain.

In general, the phylogenetic analysis of internal genes showed mostly the same results as BLAST analyses, which are presented in Table 3, except Seg-1 and -4, where due to very close identity one to another, we presented in Table 3 the next closely related sequences belonged to viruses from the global database. According to phylogenetic analysis, Israeli EHDV-6 and-1 belonged to the “Western” topotype, while EHDV-7 from 2020 belonged to the “Eastern” topotype.

## 4. Discussion

During the last fifteen years, Israeli cattle have been affected by many arboviruses for the first time. The appearance of new for the region pathogenic arboviruses has been higher than in all other countries in the Mediterranean region. Most of these viruses, which have segmented genomes, such as the Shuni virus and BTV-1, -3, -6, -8, and -9, were prominently of African origin and were seriously reassorted with the local strains [56,60,61,62], which points to their co-circulation with the local strains. 

Considering BLAST and phylogenetic analysis of Israeli EHDV-6, when six out of ten viral segments have a close relationship with Israeli EHDV-7 identified in 2006, their ancestors probably circulated in the region for a prolonged period. Considering all viral genes of Israeli EHDV-1, identified in 2016, it probably originated in Africa since nine out of ten viral segments have a high identity with African strains. Interestingly, Israeli EHDV-1 and -6 have a very close identity to Seg-1, which indicates their probable common ancestor or reassortment with the common ancestor of both viruses, which can point to probable circulation in the region different EHDVs possessing the same sequence of Seg-1. This theory can be indirectly confirmed by the evidence of the closely related sequence of Seg-1 of recently identified EHDV-8 in the region (Figure 1a). Considering the published genetic analysis of EHDV-8 [28,29] and the analysis conducted in this study, it was found that EHDV-8, registered in the Mediterranean area from 2021 to 2023, is closely related by Seg-1 and -9 to Israeli EHDV-7 from 2006, EHDV-6 from 2015, and EHDV-1 from 2016. Meanwhile, all other viral segments probably originated from different EHDVs, which were previously identified in Africa. These facts point to the appearance of a non-identified EHDV-8 of African origin in the region. To sum up, EHDV-1, -6, and -8 identified in the Mediterranean region during the last decade had regional or/and African origin.

In contrast, EHDV-7, which caused an outbreak in 2020 in Israel, possesses only “Eastern” segments and lacks “Western” segments. Therefore, it can serve as an indication of the route of introduction and timeframe of its circulation in the region. This provides us the opportunity to presume that the introduction of this virus into the country was anthropogenic because Israel imported large amounts of livestock, which can be unexpectedly infected with EHDV. This was seen in the example of imported calves from Portugal when calves were exposed to EHDV-8 (RNA of EHDV-8 was detected in the blood of these animals). Co-circulation of several serotypes in the region, processing different genotypes, can lead to the appearance of new strains with distinct clinical manifestations.

When summarizing the data on the clinical symptoms of Israeli EHDVs, it was observed that these symptoms were similar to those seen in other EHDVs worldwide. Thus, Israeli and Japanese EHDV-1 had mild clinical manifestations [26,27]. At the same time, more severe clinical signs were caused by EHDV-6 and -7 in Japan and Israel, which included fever, milk reduction, edema of the head, hyperemia, hemorrhages and lesions of the mucosal membranes and teats, lameness and stiff gate, accompanied by anorexia and respiratory distress, and sporadic death, abortion, and stillbirth [19,20,22,25]. 

Since EHDV continues to spread to new areas, preventive measures such as a restriction of the transportation of infected animals, which demands wide systematic diagnostic tests both on the exporting and importing sides, strict quarantine procedures, developing and producing vaccines, and probable medical treatment of affected animals are of high importance. This will allow for not only a decrease in economic losses caused by direct effects from the disease as death of affected animals, milk losses, abortions, slaughtering of heavily diseased animals, and expenses for symptomatic treatment but also by indirect losses as extra expenses for veterinary services and trade restrictions.

## Figures and Tables

**Table 1:** Information about Pan-EHDV RT-qPCR and EHDV isolation from cattle and different kinds of wild/zoo ill or dead animals tested in 2020–2023.

Year/Organ		Cattle			Wild Ruminants			Total	VI
		w.b.	s/l	a.f	w.b.	s/l	a.f		
2020	No. tested samples	323	19	20 (14)	0	10	1	373 (367)	
	No. of pos. samples	128	3	3 (2)	0	0	0	134 (133)	9
2021	No. tested samples	635	0	0	0	0	0	635	
	No. of pos. samples	149	0	0	0	0	0	149	
2023	No. tested samples	1067	50 (44)	26 (19)	2	30 (26)	3	1178 (1163)	
	No. of pos. samples	2	0	0	0	0	0	2	
total	No. tested samples	2025	69 (63)	46 (35)	2	40 (36)	4	2186 (2165)	9
	No. of pos. samples	279	3	3 (2)	0	0	0	285 (284)	

w.b.—whole blood samples; s/l—spleen or lung samples; a.f.—aborted fetus and newborn animals; numbers in parentheses represent number of affected fetuses/newborn animals; VI—virus isolation.

**Table 2:** Information on used primers for identification of EHDV of serotype 8.

Name of Oligo	Oligo Sequence (5′ → 3′)	Source
EHD6/8-1F	GTT AAA TTR TTC CAG GAT GGA WA	[22]
EHD6/8-250R	CAT CAT CAT AYC TCA TTA TYC CA	
		
EHD8-S2-178F	AGA GGC GCG TAA TGT TTT C	this study
EHD8-S2-522R	TGC TGA ATC ATA TCG TAA TGT A	
		
EHD8-S2-447F	CCA AAT TTG TGG AAA GCT TG	this study
EHD8-S2-705R	CGC ACT TTT GTT TGC TTA TCT TTA T	

**Table 3:** BLAST analysis of Israeli EHDV strains with global EHDV strains.

Serotype/Israeli Strain	Segment	Identity (%)	Accession Number/Serotype/Strain/Year	Country of Isolation
EHDV-6/ISR-4487/15	1	99.26	KM391733/EHDV-7/ISR2006/04/2006	Israel
	2	96.02	HM156729/EHDV-6/ALG2006/02/2006	Algeria
	3	99.37	KM391740/ EHDV-7/ISR2006/04/2006	Israel
	4	92.71	AM745020/ EHDV-4/IbAr 33853/1968	Nigeria
	5	99.46	JQ070181/EHDV-7/ISR2006/04/2006	Israel
	6	96.28	AM745072/EHDV-6/318/1983	Bahrain
	7	97.05	AY351653/EHDV/2003?	France, Reunion
	8	97.06	KM391730/EHDV-7/ISR2006/06/2006	Israel
	9	98.89	KM391747/EHDV-7/ISR2006/06/2006	Israel
	10	99.71	KM391724/ EHDV-7/ISR2006/06/2006	Israel
EHDV-1/ISR-2096/16	1	99.57	KM391733/EHDV-7/ISR2006/04/2006	Israel
	2	87.45	AM745008/EHDV-1/bAr22619/1967	Nigeria
	3	96.22	AM745019/EHDV-4/IbAr 33853/1968	Nigeria
	4	92.51	AM745020/EHDV-4/IbAr 33853/1968	Nigeria
	5	97.52	AM745021/EHDV-4/IbAr 33853/1968	Nigeria
	6	96.09	AM745012/EHDV-1/IbAr22619/1967	Nigeria
	7	97.43	AM745013/ EHDV-1/IbAr22619/1967	Nigeria
	8	95.63	AM745074/EHDV-6/318/1983	Bahrain
	9	95.59	AM745025/EHDV-4/IbAr 33853/1968	Nigeria
	10	95.05	EU928893 EHDV-3/Nigeria-ODV0001	Nigeria
	10	95.05	AM745016/EHDV-1/ IbAr22619/1967	Nigeria
EHDV-7/ISR-2262/2/20	1	96.30	LC552748/EHDV/ON-4/B/98/1998	Japan
	2	98.13	LC202943/EHDV-7/KSB-14/E/97/1997	Japan
	3	96.42	MK656455/EHDV-7/YN09-04/2013	China
	4	92.48	AM745080/EHDV-2/Ibaraki/1959	Japan
	5	97.09	MT013328/EHDV-10/JC13C644/2013	China
	6	98.52	LC202954/EHDV-7/KSB-14/E/97/1997	Japan
	7	98.60	LC552744/EHDV-7/KSB-14/E/97/1997	Japan
	8	95.75	KM509057/EHDV-2/Ibaraki BK13/1997	Japan
	9	95.20	LC552753/EHDV/ON-4/B/98/1998	Japan
	10	97.87	LC552747/EHDV-7/KSB-14/E/97/1997	Japan
EHDV-8/ISR-1692/2/23	2	99.70	OP937332/EHDV-8/*Culicoides sp*/2 TUN2022/2022	Tunisia

## Data Availability

The original contributions presented in the study are included in the article/Supplementary Material, further inquiries can be directed to the corresponding author/s.

## Supplementary Materials

The following supporting information can be downloaded at https://www.mdpi.com/article/10.3390/epidemiologia5010006/s1. Table S1: List of primers used for partial sequencing of epizootic hemorrhagic disease virus serotype 7 and bluetongue virus serotype 4; Table S2: List of sequenced Israeli EHDV strains for the present study.
