# Supplementary material for: The Molecular Epidemiology of Epizootic Hemorrhagic Disease Viruses Identified in Israel between 2015 and 2023
Source: Epidemiologia (Basel). 2024 Feb 20;5(1):90–105. doi: 10.3390/epidemiologia5010006 (PMC10885110; doi:10.3390/epidemiologia5010006)
Supplement: Supplementary file 1 [file epidemiologia-05-00006-s001.zip › epidemiologia-2797818-supplementary.pdf]

Table S1. List of primers used for partial sequencing of epizootic hemorrhagic disease virus serotype 7 and bluetongue virus serotype 4

Virus type/segment	name	Oligo sequence (5′ to 3′)	length of product	source
BTV-4,10,11,17,20,24/2	BT4-10-24-21F	ATG GAG GAR TTY GTC ATW CCW GT	329	[61]
BTV-4,10,11,17,20,24/2	BT4-10-24-330R	TCR ATR GCY CAY TTC ATC CA		[61]
Universal-EHDV/1	EHD-VP1-3F	TAA AAT GCA ATG GTC GCA ATY AC	442	this study
	EHD-VP1-426R	TRT CTC CRT ATA TYT GCA T		this study
EHDV-7/1	EHD-S1-1652F	GG AAA TAC TTC ACC CGA GTA TAA	504	this study
	EHD-S1-2136R	AAC GCC AAA TCA CTC CCA TC		this study
	EHD-S1-2275F	TCC TAT CAG AAC AAT ATG TGG G	688	this study
	EHD-S1-2941R	GCC ATC TTT GTT TCT GAT CCA A		this study
EHDV-7/2	EHD7-S2-2F	TTA AAT TGT TCC CAG GAT G	352	this study
	EHD7-S2-334R	TAA TTC TTC GAA CGC ACC CT		this study
	EHD7-S2-1585F	TCC TAA GGT TGC CTC CTT ATT	468	this study
	EHD7-S2-2031R	AAT CCG TAT AAT CAC GAG GGT C		this study
	EHD7-S2-2300F	GAT GTA TTT TTA TCA AGC GCT CAT	703	this study
	EHD7-S2-2979R	GTA AGA CTA TTG ATC CCA GTA GAC		this study
EHDV-7/3	EHD-S3-1F	GTT AAA TTT CCA GAG CGA TG	771	this study
	EHD-S3-751R	ACT CCT GCG AAA AGG TCA TAC		this study
	EHD-S3-1145F	TTT AAT GTT TAC AGC GGG AC	1107	this study
	EHD-S3-2229R	TCA TAT GTA TAC TGC ACC ACT CG		this study
	EHD-S3-2422F	CGA GTG AGA AAG GTG GGC TA	348	this study
	EHD-S3-2748R	GTA AGT GTA TTA CCC AGT GCC T		this study
EHDV-7/4	EHD-S4-1F	GTT AAA ACA TGC CGG AGC C	363	this study
	EHD-S4-363R	ATG CGT AGC TAC GTA GGA TGT C		this study
	EHD-S4-563F	TGC CAT CTA TAG CTG TGA AGA	1000	this study
	EHD-S4-1543R	GCA TAG CGA TAT GTC AGG AA		this study
	EHD-S4-1710F	TGG TGG TTT AAA ATG ATA TTA GA	267	this study
	EHD-S4-1956R	TAG GTG TAA CAT GCA GGC CTT		this study
EHDV-7/8	EHD-S8-213F	CGT ATC GCA TTC AGG ATG G	735	this study
	EHD-S8-926R	CGA GCA TTG ACA ATC GTT CAT C		this study
Universal-EHDV/9	EHD-VP6-4F	AAA AAT TGC GCA TGT CAG C	409	this study
	EHD-VP6-394R	CTT CCT CCT CCC GTC CCA T		this study
EHDV-7/9	EHD-S9-1F	GTT AAA AAA TTG CGC ATG TCA	307	this study

	EHD-S9-287R	GTT CTC CAG ATC GAT TAT CGC		this study
Universal-BTV-EHDV/10	EHD-S10-1F	GTT AAA AAG AGG TTG GTG CC	318	this study
EHDV-7/10	EHD-S10-297R	TCA ACC ATC ACD CCA TTA TGT T		this study
Universal-EHDV/10	BT-EHD-S10R	ACCCTCCCCCGYTAKACARC	787	[62]

Table S2. List of sequenced Israeli EHDV strains for the present study

serotype	year	segment/ length<br>strain	1/3942	2/3002	3/2768	4/1983-1984	5/1770	6/1641	7/1162	8/1186	9/1074-1140	10/810
6	2015	ISR-4487/15	OM502363	OM502364	OM502365	OM502366	OM502367	OM502368	OM502369	OM502370	OM502371	OM502372
			12-3920	17-2932	18-2717	1-1935	33-1682	28-1611	18-1067	20-1141	15-1094	21-707
1	2016	ISR-2096/16	OM502373	OM502374	OM502375	OM502376	OM502377	OM502378	OM502379	OM502380	OM502381	OM502382
			12-3920	17-2931	18-2717	9-1943	33-1688	28-1611	18-1067	20-1141	15-1094	21-707
7	2020	ISR-2262/2/20	OR602552	OR602553	OR602554	OR602555	OR602556	OR602557	OR602558	OR602559	OR602560	OR602561
			1-3942	2-2995	4-2768	1-1981	13-1766	12-1632	10-1150	10-1185	1-1062	1-797
7	2020	ISR-2556/20	OR774975	no	no	no	no	no	no	no	OR774976	OR774977
			2-444								8-390	62-701
7	2020	ISR-2647/20	OR774978	no	no	no	no	no	no	no	OR774979	no
			2-444								3-389	
7	2020	ISR-2262/3/20	OR774980	no	no	no	no	no	no	no	OR774981	OR774982
			24-430								14-389	35-691
8	2023	ISR-1692/2/23	no	OR636216	no	no	no	no	no	no	no	no
				23-698								
8	2023	ISR-1692/9/23	no	OR636217	no	no	no	no	no	no	no	no
				23-544								

Upper rows provide accession numbers of sequenced regions. Lower rows show sequences regions.
